# Treatment of Pneumonia

**Published:** 1862-03

**Authors:** 


### SELECTIONS.

Treatment of Pneumonia.—The Sanitary Committee, on the Treatment of Pneumonia, consisting of Austin Flint, M. D., Chairman; A. Clark, M. D., John T. Metcalfe, M. D., Benj. W. M'Cready, M. D., conclude their report, as follows:

"The following propositions are submitted, embodying the practical views which have been presented respecting the management of pneumonia:

1.—Uncomplicated pneumonia, limited to one lobe, in general, does not claim active treatment of any kind—simple, paliative remedies and hygienic measures being alone required.

2.—Blood-letting and other antiphlogistic measures, with a view of subduing the inflammation, are not warranted by a sufficient probability of success, and, if resorted to for this purpose, will be likely in many cases to do harm.

3.—Blood-letting is useful, not by a direct effect on the local affection, but indirectly by diminishing the intensity of the symptomatic febrile movement. It is admissible only in cases characterized by intensity of the febrile movement, when the affection is said to be sthenic, and only in the first stage of the affection.

4.—In the cases to which blood-letting, if employed at all, should be restricted, the good effects may generally be obtained by saline purgatives, together with sedative remedies, such as the preparations of antimony and the veratrum viride.

5.—The remedies just named are indicated only in the cases referred to. Given in cases indiscriminately, and carried to an injudicious extent, they may do much harm. They should be used with great circumspection, and rarely after the first stage of the disease. It is never advisable to push them so far as to occasion distressing nausea or vomiting, and enfeeble the heart's action.

6.—Acute pain, depending on co-existing pleurisy, does not call for general blood-letting. Dry or wet cupping, fomentations, and stimulating applications to the chest are useful, and, if not effectual, opium may be given sufficiently to relieve this symptom. The oiled muslin jacket, to be worn during the disease, is to be recommended.

7.—The combination of intermitting fever and pneumonia calls for the prompt use of quinia in sufficient doses to arrest as speedily as possible the paroxysmal affection. Small or moderate doses of this remedy should be given in malarious regions, and to patients who are subject to intermitting fever, in order to prevent the development of intermitting fever and to obviate the unfavorable influence of the malarious cachexia. The remedy should be continued during the progress of the disease.

8.—Antimonial preparations, mercury, blisters, and expectorants are not called for with a view to promote resolution of the pulmonary affection. There are not sufficient grounds for the belief that they hasten the removal of the exudation, and, if not useful, they must be injurious. There are no remedies to be employed specially for this object.

9.—In severe cases of pneumonia, after the disease has advanced to the second stage, the most important object of treatment generally is to support the powers of life, to obviate the tendency to death by asthenia, and to carry the patient safely through the disease.

10.—The supporting treatment consists of tonic remedies, alcoholic stimulants, and nutritious food. These are to be combined, in order to render the supporting treatment efficient.

11.—Alcoholic stimulants may be given without fear of affecting unfavorably the local affection. They should be given so soon, at least, as the heart's action and other symptoms afford evidence of any failure of the vital powers. They are to be given more or less freely, according to the danger from asthenia, the degree of tolerance, and the apparent effect.

They are not to be given as a matter of course, but only when indicated, and the quantity given is to be determined by the exercise of care and judgment.

12.—A supporting diet embraces the animal essences, milk, and farinaceous articles. There is no risk in encouraging the patient to take nutritious food at any time during the progress of the disease; and there is reason to believe that danger from exhaustion may be forestalled by alimentation, together with the early employment of tonic remedies and alcoholic stimulants.

13.—Purgatives, after the first stage, are not indicated, save when there is inconvenience from fœcal accumulation, and then the mildest remedies which will effect the object are to be preferred.

14.—Opium given, not to relieve pain or allay cough, but to tranquilize, promote sleep, and render the system more tolerant of the local affection, is a valuable remedy in pneumonia. It is indicated by unusual disturbance of the circulation and nervous system, and its good effect is shown by a marked improvement in all the symptoms. This remedy does not retard the resolution of the local affection. It conduces frequently to improvement as regards delirium.

15.—Soothing and supporting measures are especially called for in cases of pneumonia distinguished as asthenic and typhoid, and when pneumonia occurs as a complication of the eruptive and continued fevers.

16.—The occurrence of pericarditis as a complication is an additional reason for the supporting treatment.

17.—In convalescence from pneumonia there is not much, if any, danger of relapse, and the recovery is more rapid if a substantial diet be allowed and the patient permitted early to sit up."

